# La-doped BaSnO3 for electromagnetic shielding transparent conductors

**DOI:** 10.1186/s40580-023-00397-z

**Published:** 2023-10-28

**Authors:** Jingyeong Jeon, Youngkyoung Ha, Judith L. MacManus-Driscoll, Shinbuhm Lee

**Affiliations:** 1 Department of Physics and Chemistry, Department of Emerging Materials Science, DGIST, Daegu, 42988 Republic of Korea; 2 Department of Materials Science and Metallurgy, University of Cambridge, 27 Charles Babbage Road, Cambridge, CB3 0FS UK

**Keywords:** Transparent conductors, Electromagnetic shielding, Ba_1−*x*_La_*x*_SnO_3_, MgAl_2_O_4_, Templated epitaxy, Single crystallinity, Doping dependence

## Abstract

In this work, we find that La-doped BaSnO_3_ (BLSO) is shown to be a promising electromagnetic shielding transparent conductor. While films grown on industrially practical optoelectronic MgAl_2_O_4_ substrates have higher sheet resistance by three orders of magnitude than in previous reports, we show how to recover the sheet resistance close to the single-crystal level by use of an MgO template layer which enables high quality (001)-oriented BLSO epitaxial film growth on (001) MgAl_2_O_4_. There is a positive correlation between crystallinity and conductivity; high crystallinity minimizes scattering of free electrons. By applying this design principle to 5–20% doped films, we find that highly crystalline 5% La-doped BLSO films exhibit low sheet resistance of ~ 8.7 Ω ▯ ^−1^, high visible transmittance of ~ 80%, and high X-band electromagnetic shielding effectiveness of ~ 25.9 dB, thus outperforming transparent conducting oxides films of Sn-doped In_2_O_3_ and SrMoO_3_.

**Graphical Abstract:**

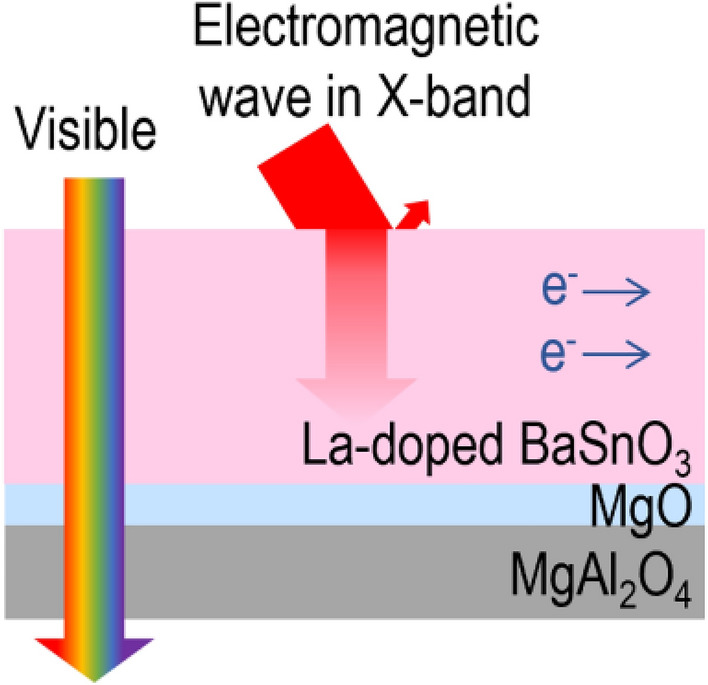

**Supplementary Information:**

The online version contains supplementary material available at 10.1186/s40580-023-00397-z.

## Introduction

Transparent conductors with electromagnetic shielding capabilities (TC-EMS) have attracted great interest because radiation damages human health and it causes sensitive electronic systems to malfunction [1–9]. Interest in TC-EMS has skyrocketed for new applications working in extreme environments, including invisible circuits, smart windows, transparent solar cells, and protective window coatings. Such demands impose stringent requirements on new stable transparent materials with high electromagnetic shielding efficiency [10–12]. To achieve the ideal TC-EMS material, a low sheet resistance (< 200 Ω ▯^−^^1^), high electromagnetic shielding capability (> 20 dB at 10 GHz in the X-band), and high-level visible transmittance (> 50% at a wavelength of 550 nm) are required. In addition to this, for many emerging applications thermal stability above 300 °C is needed. Metal films have been widely studied for electromagnetic shielding, but they usually show weak light transmittance. Irrespective of the light transmittance, standard metal and carbon meshes are insufficiently conductive, are susceptible to corrosion, mechanical weakness, and are difficult to shape. Two-dimensional materials are another contender system for TC-EMS. While they have an exponential increased level of transmittance compared to metal films, they tend to be mechanically weak and are difficult to achieve in large area via mass production.

La-doped BaSnO_3_ (BLSO) is a well-known wide bandgap (3.3–4.1 eV) transparent conductor with chemical and mechanical robustness [13–28]. The established charge transfer mechanism is from the valence band of O 2*p* orbitals to the conduction band of the Sn 5* s* orbital) [13, 15]. Aliovalent cation (e.g., La^3+^) doping renders Ba^2+^Sn^4+^O_3_ conductive, similar to prototypical wide-bandgap Sn^4+^-doped ${\mathrm{In}}_{2}^{3+}{\mathrm{O}}_{3}$ [29, 30]. BLSO exhibits high electron mobility in single crystals (~ 250 cm^2^ V^–1^ s^–1^) [13, 15] and films (~ 100 cm^2^ V^–1^ s^−^^1^) [15, 22] at room temperature; the straight O–Sn–O connectivity and large Sn 5* s* orbital in the cubic perovskite structure creates a dispersive conduction band with a small effective mass. BLSO has been shown to be thermally stable in air above 530 °C [14], while SrMoO_3_ films lose metallicity above 450 °C because of over-oxidization [12]. The films can be coated over wide areas using a simple one-step process. However, irrespective of such excellent properties, few studies have used BLSO for TC-EMS applications. 

In this work, we grow BLSO films on industrially practical optoelectronic MgAl_2_O_4_ substrates for TC-EMS. We show that metallic sheet resistances at the single-crystal level can be achieved by inserting an MgO template layer between the film and the substrate. At the same time, the films have high visible transmittance and X-band shielding effectiveness (SE). On the other hand, when the MgO template layer is not used, films have three orders of magnitude higher resistances owing to defect scattering. X-ray diffraction (XRD) and scanning transmission electron microscopy (STEM) studies revealed that our templated-epitaxy-approach induced a strong enhancement of film crystallinity. Given the positive correlation between conductivity and crystallinity, Ba_0.95_La_0.05_SnO_3_ films yield the best-performing TC-EMS among reported materials.

Hereafter, we use the simpler form $(100\times x)\%-{\mathrm{BLSO}}_{\mathrm{substrate}}^{\mathrm{template\,layer}}$ for Ba_1−*x*_La_*x*_SnO_3_ (e.g., $5\% - {\text{BLSO}}_{{{\text{MgAl}}_{2} {\text{O}}_{4} }}^{{{\text{MgO}}}}$ for the Ba_0.95_La_0.05_SnO_3_ films on MgAl_2_O_4_ with an MgO template layer).

## Single-crystal-level sheet resistance of La-doped BaSnO_3_ (BLSO) films on (001) MgAl_2_O_4_ with an MgO template layer

MgAl_2_O_4_ is an industrially practical substrate for applications in microwave acoustics and optoelectronics and hence is a good substrate for BLSO films for TC-EMS. Most studies on BLSO films report the properties of epitaxial films grown on cubic substrates (e.g., SrTiO_3_, KTaO_3_, and MgO) [14–22, 24–28] but growth on MgAl_2_O_4_ has not been reported previously. The similar cubic structures of BLSO films and SrTiO_3_, KTaO_3_, and MgO substrates enable cube-on-cube epitaxial growth. This is because of the moderate lattice mismatch ($=\frac{{a}_{\mathrm{substrate}}-{a}_{\mathrm{film}}}{{a}_{\mathrm{film}}}\times 100$) (between −5.22% and +1.94%) along the [100] BLSO || [100] substrate, where *a*_substrate_ denotes the lattice parameters of SrTiO_3_ (*a* = *b* = *c* = 3.905 Å), KTaO_3_ (3.99 Å) and MgO (4.20 Å), and *a*_film_ is the lattice parameter of BaSnO_3_ (4.12 Å). However, SrTiO_3_ and KTaO_3_ are quite expensive, and MgO tends to absorb water [31] and suffers from poor quality [32]. It was quite surprising that most researches are still limited to the industrially impractical substrates of SrTiO_3_, KTaO_3_, or MgO although ten years have passed after the first introduction of BLSO [13–16]. Now, it is time to find industrially practical new substrates for commercial optoelectronic applications of BLSO. MgAl_2_O_4_ exhibits chemical, thermal, and mechanical stability and is much cheaper than perovskite substrates.

**Fig. 1:**
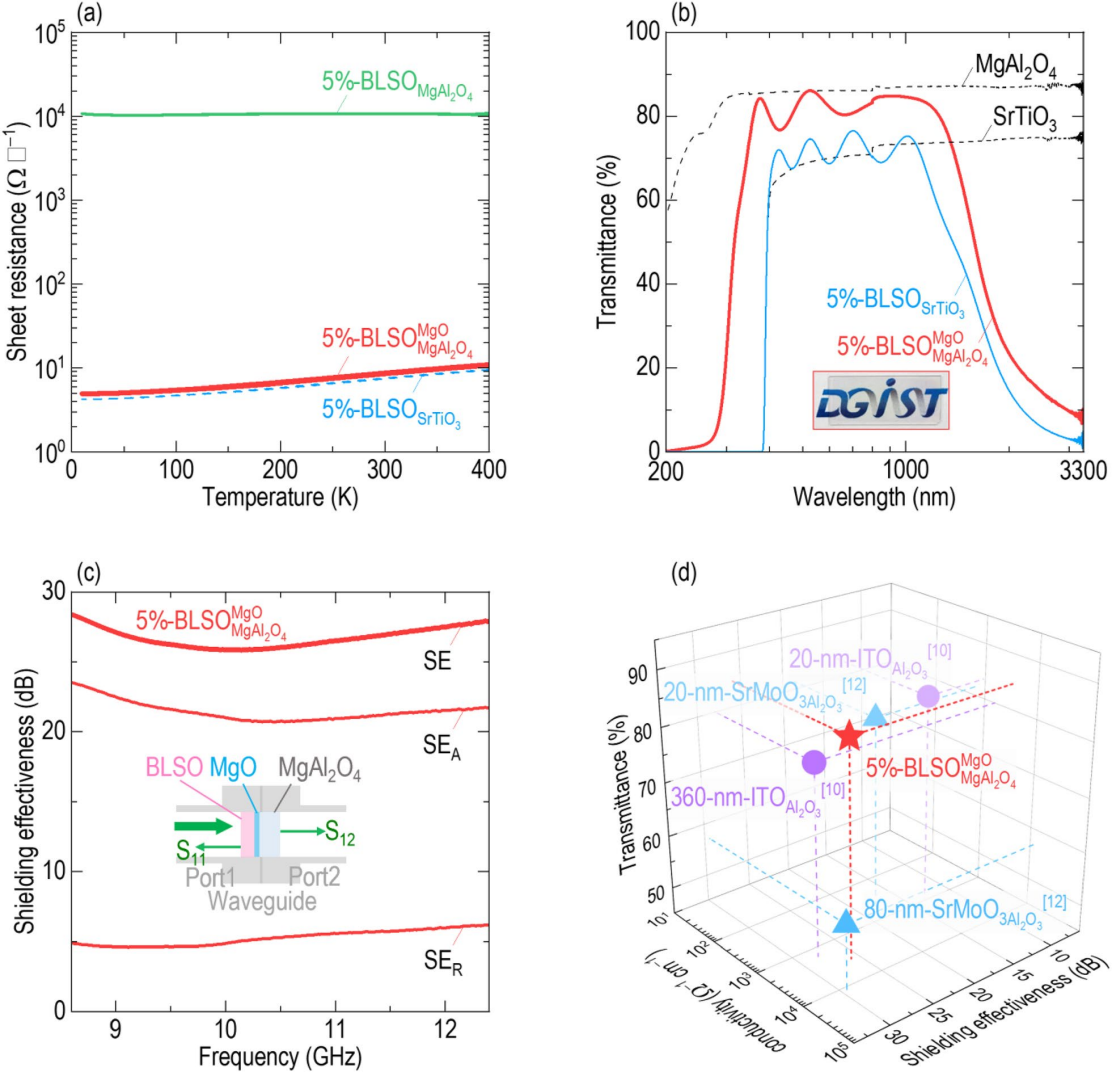
Ba_1−*x*_La_*x*_SnO_3_ (BLSO) films grown on (001)-oriented MgAl_2_O_4_ with an MgO template layer to fabricate transparent conductors with electromagnetic shielding capabilities (TC-EMS). For convenience, we use the simpler form of ${(100\times x)\mathrm{\%}-\mathrm{BLSO}}_{\mathrm{substrate}}^{\mathrm{template\,layer}}$. **a** The sheet resistance of $5\%-{\mathrm{BLSO}}_{{\mathrm{MgAl}}_{2}{\mathrm{O}}_{4}}^{\mathrm{MgO}}$ is lower by three orders of magnitude than that of ${\mathrm{BLSO}}_{{\mathrm{MgAl}}_{2}{\mathrm{O}}_{4}}$, and thus comparable to that of single-crystalline ${\mathrm{BLSO}}_{{\mathrm{SrTiO}}_{3}}$. **b** The $5\%-{\mathrm{BLSO}}_{{\mathrm{MgAl}}_{2}{\mathrm{O}}_{4}}^{\mathrm{MgO}}$ exhibits high transmittance (> 85%) in the visible region; the “DGIST” logo can be seen through the $5\%-{\mathrm{BLSO}}_{{\mathrm{MgAl}}_{2}{\mathrm{O}}_{4}}^{\mathrm{MgO}}$. The transmittance in the infrared region was suppressed by the free electron response. The fundamental absorption edge of $5\%-{\mathrm{BLSO}}_{{\mathrm{MgAl}}_{2}{\mathrm{O}}_{4}}^{\mathrm{MgO}}$, at which the transmittance drops sharply at ultraviolet wavelengths, lies at a shorter wavelength of ~ 300 nm than ~ 400 nm of ${\mathrm{BLSO}}_{{\mathrm{SrTiO}}_{3}}$. The dashed lines indicate the transmittances of the MgAl_2_O_4_ and SrTiO_3_ substrates. **c** The $5\%-{\mathrm{BLSO}}_{{\mathrm{MgAl}}_{2}{\mathrm{O}}_{4}}^{\mathrm{MgO}}$ shielding effectiveness (SE) is ~ 25.9 dB at 10 GHz. SE_A_ and SE_R_ represent the wave transmitted through the film and MgAl_2_O_4_ substrate, and the wave reflected from the BLSO film, respectively. The films exhibit an SE_A_ of ~ 21.0 dB, which is larger than the SE_R_ of ~ 4.9 dB at 10 GHz. **d** The $5\%-{\mathrm{BLSO}}_{{\mathrm{MgAl}}_{2}{\mathrm{O}}_{4}}^{\mathrm{MgO}}$ thus shows superior conductivity, transmittance, and SE than the potential TC-EMS materials Sn-doped In_2_O_3_ (ITO) [10] and SrMoO_3_. [12]

Unexpectedly, the sheet resistance of BLSO films directly grown on (001)-oriented MgAl_2_O_4_ was significantly higher by three orders of magnitude than that of single-crystalline films on (001) SrTiO_3_. Figure 1a shows the temperature dependence of the sheet resistances of $5\% - {\text{BLSO}}_{{{\text{MgAl}}_{2} {\text{O}}_{4} }}^{{{\text{MgO}}}}$, $5\% - {\text{BLSO}}_{{{\text{MgAl}}_{2} {\text{O}}_{4} }}$ and $5\% - {\text{BLSO}}_{{{\text{SrTiO}}_{3} }}$. The sheet resistances of ${\mathrm{BLSO}}_{{\mathrm{MgAl}}_{2}{\mathrm{O}}_{4}}$ and ${\mathrm{BLSO}}_{{\mathrm{SrTiO}}_{3}}$ were ~ 11,000 Ω ▯^−^^1^ and ~ 7.6 Ω ▯^−^^1^, respectively, at room temperature. The sheet resistance of ${\mathrm{BLSO}}_{{\mathrm{MgAl}}_{2}{\mathrm{O}}_{4}}$ exhibited weak temperature dependence, whereas that of ${\mathrm{BLSO}}_{{\mathrm{SrTiO}}_{3}}$ increased with increasing temperature (Additonal file 1: Figure S1 for the normalized sheet resistances), indicating the insulating and metallic ground states, respectively. Such poor conductivity could hinder the further development of BLSO for optoelectronic devices.

However, after placing an MgO template layer, we found that the sheet resistance of ${\mathrm{BLSO}}_{{\mathrm{MgAl}}_{2}{\mathrm{O}}_{4}}^{\mathrm{MgO}}$ recovered to ~ 8.7 Ω ▯^−1^, comparable to that of single-crystalline ${\mathrm{BLSO}}_{{\mathrm{SrTiO}}_{3}}$. The conductivity, carrier mobility, and density of ${\mathrm{BLSO}}_{{\mathrm{MgAl}}_{2}{\mathrm{O}}_{4}}^{\mathrm{MgO}}$ were comparable to reports in single crystals, ${\mathrm{BLSO}}_{{\mathrm{SrTiO}}_{3}}$, and ${\mathrm{BLSO}}_{\mathrm{MgO}}$ (Additional file 1: Figure S2, Table S1 for comparison of conductivity, carrier mobility, and density among the reports of BLSO), indicating that BLSO films could showcase excellent conducting properties even on the industrially practical MgAl_2_O_4_ substrate with assistance of MgO template layer. It should be noted that the sheet resistance of ${\mathrm{BLSO}}_{{\mathrm{MgAl}}_{2}{\mathrm{O}}_{4}}^{\mathrm{MgO}}$ was smaller than 18–97 Ω ▯^–1^ on 40–360-nm-thick Sn-doped In_2_O_3_ films [10] and 10–38 Ω ▯^–1^ on 45–80-nm-thick SrMoO_3_ films [12]. The availability of highly conducting ${\mathrm{BLSO}}_{{\mathrm{MgAl}}_{2}{\mathrm{O}}_{4}}^{\mathrm{MgO}}$ motivated us to measure the transmittance, and X-band SE thereof to evaluate the possibility of use for TC-EMS.

## High visible transmittance and high electromagnetic shielding of conducting BLSO

Figure 1b shows the transmittance of $5\% - {\text{BLSO}}_{{{\text{MgAl}}_{2} {\text{O}}_{4} }}^{{{\text{MgO}}}}$ from 200 to 3,300 nm. For comparison, we also measured the transmittance of $5\% - {\text{BLSO}}_{{{\text{SrTiO}}_{3} }}$. ${\mathrm{BLSO}}_{{\mathrm{MgAl}}_{2}{\mathrm{O}}_{4}}^{\mathrm{MgO}}$ showed high transmittance (~ 85%) over the visible wavelength range of 400–1000 nm and was also transparent at ~ 300 nm (see the “DGIST” logo through the BLSO film). The oscillating transmittance in the visible region was likely attributable to interference between light reflected from the film and the substrate for 440-nm-thick BLSO films [33]. The visible transmittance of BLSO was higher than ~ 80% of 40–360-nm-thick Sn-doped In_2_O_3_ films [10] and ~ 60% of 45–80-nm-thick SrMoO_3_ films [12]. In these contexts, ${\mathrm{BLSO}}_{{\mathrm{MgAl}}_{2}{\mathrm{O}}_{4}}^{\mathrm{MgO}}$ was superior to ${\mathrm{BLSO}}_{{\mathrm{SrTiO}}_{3}}$, whose transmittance was ~ 70% in the visible and abruptly decreased below ~ 400 nm.

MgAl_2_O_4_ and MgO have much larger bandgaps (7.6–7.8 eV) [34] than the 3.2 eV of SrTiO_3_, and thus did not contribute to the ultraviolet absorption edge of $5\% - {\text{BLSO}}_{{{\text{MgAl}}_{2} {\text{O}}_{4} }}^{{{\text{MgO}}}}$. Therefore, we estimated that the bandgap of Ba_0.95_La_0.05_SnO_3_ films was ~ 4.1 eV, similar to that of single crystals [13]. The transmittance of both ${\mathrm{BLSO}}_{{\mathrm{MgAl}}_{2}{\mathrm{O}}_{4}}^{\mathrm{MgO}}$ and ${\mathrm{BLSO}}_{{\mathrm{SrTiO}}_{3}}$ was suppressed at infrared wavelengths (> 1000 nm) because of the free electron response typically shown in metals.

Figure 1c shows SE of $5\% - {\text{BLSO}}_{{{\text{MgAl}}_{2} {\text{O}}_{4} }}^{{{\text{MgO}}}}$ over the X-band frequency range (8.5–12.5 GHz) overlapped with the radio wave (10^4^–10^10^ Hz) and microwave (10^9^–10^12^ Hz) regimes. The schematic inset shows the coaxial transmission line method used to measure the SE. The high SE of ~ 25.9 dB at 10 GHz was comparable to those of 40–360-nm-thick Sn-doped In_2_O_3_ films (16.1–28.1 dB) [10], 45–80-nm-thick SrMoO_3_ films (27.3–29.4 dB) [12] and metal films, metal meshes, and two-dimensional materials [1–9]. We resolved the SE (= SE_A_ + SE_R_) into SE_A_ and SE_R_ (denoting shielding by absorption through the BLSO film and reflection from the film, respectively). The larger SE_A_ of ~ 21.0 dB at 10 GHz than the SE_R_ of ~ 4.9 dB indicated that absorption was the dominant mechanism of electromagnetic shielding. Thus, ${\mathrm{BLSO}}_{{\mathrm{MgAl}}_{2}{\mathrm{O}}_{4}}^{\mathrm{MgO}}$ is a promising TC-EMS material for stealth technologies.

## Promising BLSO films for electromagnetic shielding transparent conductors

Figure 1d shows the superior sheet resistance, transmittance, and SE of $5\% - {\text{BLSO}}_{{{\text{MgAl}}_{2} {\text{O}}_{4} }}^{{{\text{MgO}}}}$ compared to those of Sn-doped In_2_O_3_ and SrMoO_3_. Given their high conductivity and SE, BLSO films provide much higher transmittance than other potential electromagnetic shielding materials (Additional file 1: Table S2 for dopant concentration in BaSnO_3_, film thickness, substrate, and deposition techniques in reports). It is clear that BLSO films are suitable for TC-EMS because they have low sheet resistance, high visible transmittance, high X-band SE, chemical and mechanical stability, and the possibility to be fabricated over wide areas. In addition, the use of MgAl_2_O_4_ will reduce the costs of emerging TC-EMS applications, compared to the perovskite substrates.

## Enhanced crystallinity of BLSO films on (001) MgAl_2_O_4_ using an atomically matched MgO template layer

**Fig. 2:**
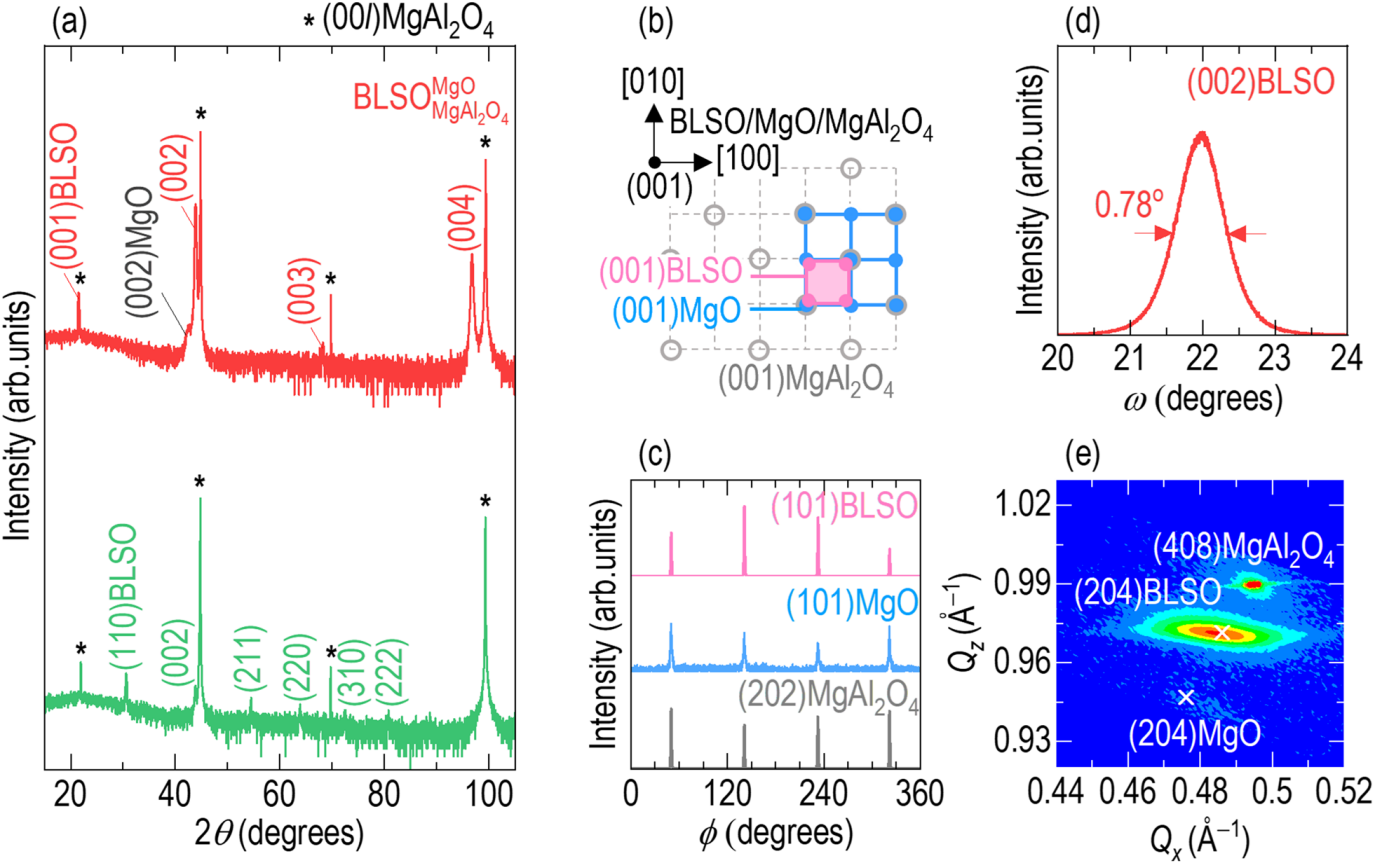
Epitaxial stabilization of Ba_0.95_La_0.05_SnO_3_ films on (001)MgAl_2_O_4_ with an MgO template layer. **a** The X-ray diffraction (XRD) *θ−*2*θ* scan of ${\mathrm{BLSO}}_{{\mathrm{MgAl}}_{2}{\mathrm{O}}_{4}}^{\mathrm{MgO}}$ exhibits (001), (002), (003), and (004) diffraction peaks of BLSO and MgO, indicating the formation of (001)-oriented BLSO epitaxial films on (001)MgAl_2_O_4_ with a (001)MgO template layer. The XRD *θ−*2*θ* scan of ${\mathrm{BLSO}}_{{\mathrm{MgAl}}_{2}{\mathrm{O}}_{4}}$ indicates the formation of mixed crystallographic orientations of BLSO. The asterisks indicate the (002), (004), (006), and (008) diffraction peaks of MgAl_2_O_4_. As schematically shown in (**b**), **c** the XRD *ϕ* scans of (101)BLSO and (101) MgO show four diffraction peaks at the same *ϕ* angles as those of (202)MgAl_2_O_4_, indicating four-fold symmetrical in-plane matching. **d** The low full-width at half-maximum of 0.78° in the XRD *ω*-scans of (002)BLSO indicates high crystallinity of BLSO epitaxial films with an MgO template layer. **e** Reciprocal space mapping indicates the absence of strain in the BLSO film and MgO template layer on the MgAl_2_O_4_ substrate, given that the (204)BLSO and (204)MgO peaks are near the *Q*_*x*_- and *Q*_*z*_-values of their bulks (× symbols)

At first glance, the lack of metallic behavior in $5\% - {\text{BLSO}}_{{{\text{MgAl}}_{2} {\text{O}}_{4} }}^{{{\text{MgO}}}}$ was surprising since we expected single-crystalline growth of (001)-oriented BLSO epitaxial films on (001) MgAl_2_O_4_. However, as shown by the XRD *θ−*2*θ* scan of ${\mathrm{BLSO}}_{{\mathrm{MgAl}}_{2}{\mathrm{O}}_{4}}$ (bottom panel of Fig. 2a), there were many weak peaks of (110), (211), (220), (310), and (222) BLSO, as well as the (002) BLSO peak, indicating the polycrystalline nature of the films (Additional file 1: Figure S3 for nanoscopic investigation of the crystal structure of $5\%-{\mathrm{BLSO}}_{{\mathrm{MgAl}}_{2}{\mathrm{O}}_{4}}$). As the cubic structures of perovskite BLSO (*a* = *b* = *c* = 4.12 Å) and spinel MgAl_2_O_4_ (8.09 Å) are similar with only moderate lattice mismatch ($=\frac{{a}_{\mathrm{substrate}}-2\times {a}_{\mathrm{film}}}{2\times {a}_{\mathrm{film}}}\times 100$) of −1.8% along [100]BLSO || [100]MgAl_2_O_4_, the MgAl_2_O_4_ substrates may be expected to enable cube-on-cube epitaxial growth of BLSO films. Also, the thermal expansion coefficients of BLSO (9.3 × 10^−6^ °C^−1^) [35] and MgAl_2_O_4_ (7.5 × 10^−6^ °C^−1^) are similar. However, despite the small lattice mismatch and similar coefficients of thermal expansion, the different crystal structures between perovskite BLSO and spinel MgAl_2_O_4_ would mean poor atomic matching at the BLSO and MgAl_2_O_4_ (Fig. 2b) interface. This would lead to higher interfacial energy rendering several different BLSO crystal orientations to have similar stabilities on the MgAl_2_O_4_ surface [36].

Recovery of sheet resistance to the single-crystal level was attributable to epitaxial growth of BLSO films on MgAl_2_O_4_ with an MgO template layer. The top panel of Fig. 2a shows an XRD *θ*–2*θ* scan of $5\% - {\text{BLSO}}_{{{\text{MgAl}}_{2} {\text{O}}_{4} }}^{{{\text{MgO}}}}$. As well as the (004) peak at 44.8° and (008) peak at 99.4° of MgAl_2_O_4_, there were very strong peaks of BLSO at 2* θ* = 21.5°, 43.9°, 68.2°, and 96.8°, arising from diffraction by the (001), (002), (003), and (004) planes of BLSO, respectively. Although the similar lattice parameters of cubic BLSO (4.12 Å) and MgO (4.20 Å) made it difficult to separate their XRD peaks, we found a small peak of (002)MgO at 42.6°. Therefore, the XRD *θ−*2*θ* scan indicated (001) BLSO epitaxial film growth on (001) MgAl_2_O_4_ with assistance from the (001)-oriented epitaxial growth of the MgO template layer. We speculate that the 1000-fold rise in conductivity of Ba_0.95_La_0.05_SnO_3_ epitaxial films on (001) MgAl_2_O_4_ is attributable to minimal scattering of free electrons by the enhanced crystallinity promoted by the MgO template layer. We explore this further below.

Epitaxial growth in the presence of the MgO template layer is understandable based on the similar atomic arrangements of MgO and BLSO which enables single-crystal growth of the BLSO epitaxial films on the MgO-templated MgAl_2_O_4_. The lattice mismatch ($=\frac{{a}_{\mathrm{substrate}}-2\times {a}_{\mathrm{template\,layer}}}{2\times {a}_{\mathrm{template\,layer}}}\times 100$) between MgAl_2_O_4_ and MgO (4.20 Å) is −3.7%, and that between MgO and BLSO is + 1.9%. Thus, the template layer does not mitigate the mismatch of −1.8% between BLSO and MgAl_2_O_4_. On the other hand, the continuous atomic arrangement of MgO and BLSO enables cube-on-cube epitaxial growth of BLSO films on the MgO template layer with the epitaxial relationship (001)BLSO/MgO || (001)MgAl_2_O_4_ and [100]BLSO/MgO || [100]MgAl_2_O_4_ (Fig. 2b). This relationship was supported by four strong diffraction peaks in the XRD *ϕ* scans of (101) BLSO and (101) MgO, at the same *ϕ* angles as those of (202) MgAl_2_O_4_ (Fig. 2c). The full-width at half-maximum (FWHM) of the (002) BLSO peaks in the *ω* scans was as small as 0.78° (Fig. 2d), indicating minimal mosaic spread of BLSO films by templated epitaxy. Reciprocal space mapping revealed that the (204) BLSO and (204) MgO peaks were near the *Q*_*x*_- and *Q*_*z*_-values of their bulks (× symbols in Fig. 2e), indicating that the BLSO film and MgO template layer were strain-free on the MgAl_2_O_4_ substrate.

**Fig. 3:**
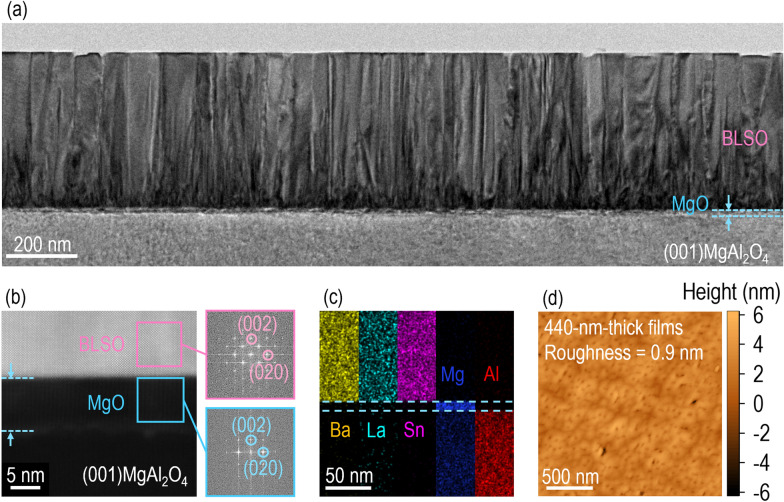
Nanoscopic investigation of the crystal structure of $5\%-{\mathrm{BLSO}}_{{\mathrm{MgAl}}_{2}{\mathrm{O}}_{4}}^{\mathrm{MgO}}$. **a** The cross-sectional transmission electron microscopic image clearly shows the BLSO film, MgO template layer, and MgAl_2_O_4_ substrate. **b** Given the sharp interfaces, fast Fourier transform images of selected areas indicate (001)-oriented epitaxial growth of the BLSO film and MgO template layer. **c** Energy-dispersive X-ray spectroscopy reveals negligible intermixing of Ba (yellow), La (green), Sn (purple), Mg (blue), and Al (red) atoms among the layers. **d** Atomic force microscopy reveals the flat surface (roughness ~ 0.9 nm) of a 440-nm-thick BLSO film

To obtain further insight into the crystallinity of $5\% - {\text{BLSO}}_{{{\text{MgAl}}_{2} {\text{O}}_{4} }}^{{{\text{MgO}}}}$, we used transmission electron microscopy to acquire cross-sectional images of the epitaxial films (Fig. 3a). The 440-nm-thick BLSO films had very flat surfaces. Over a 2.2-μm-wide region, we could distinguish the BLSO film, MgO template layer, and MgAl_2_O_4_ substrate using the dark and bright regions (where brightness reflects atomic number). The STEM image (Fig. 3b) shows that, at the interfaces of the $5\% - {\text{BLSO}}_{{{\text{MgAl}}_{2} {\text{O}}_{4} }}^{{{\text{MgO}}}}$heterostructure, there was minimal atomic intermixing among the BLSO film, MgO template layer, and MgAl_2_O_4_ substrate. Also, a 10-nm-thick MgO template layer enabled BLSO epitaxial growth. Energy-dispersive X-ray spectroscopy indicated uniform distributions of Sn (purple), Mg (blue), and Al (red) atoms over the entire area, reflecting minimal atomic intermixing among the film, template layer, and substrate (Fig. 3c). The surface roughness as determined using atomic force microscopy confirmed that the 440-nm-thick $5\%-{\mathrm{BLSO}}_{{\mathrm{MgAl}}_{2}{\mathrm{O}}_{4}}^{\mathrm{MgO}}$ was extremely small (~ 0.9 nm) (Fig. 3d). This roughness is similar to the ~ 0.78 nm of $5\%-{\mathrm{BLSO}}_{{\mathrm{SrTiO}}_{3}}$ (Additional file 1: Figure S4 for the flat surface roughness).

## Optimization of La concentration in BLSO epitaxial films on (001) MgAl_2_O_4_ for fabrication of electromagnetic shielding transparent conductors

**Fig. 4:**
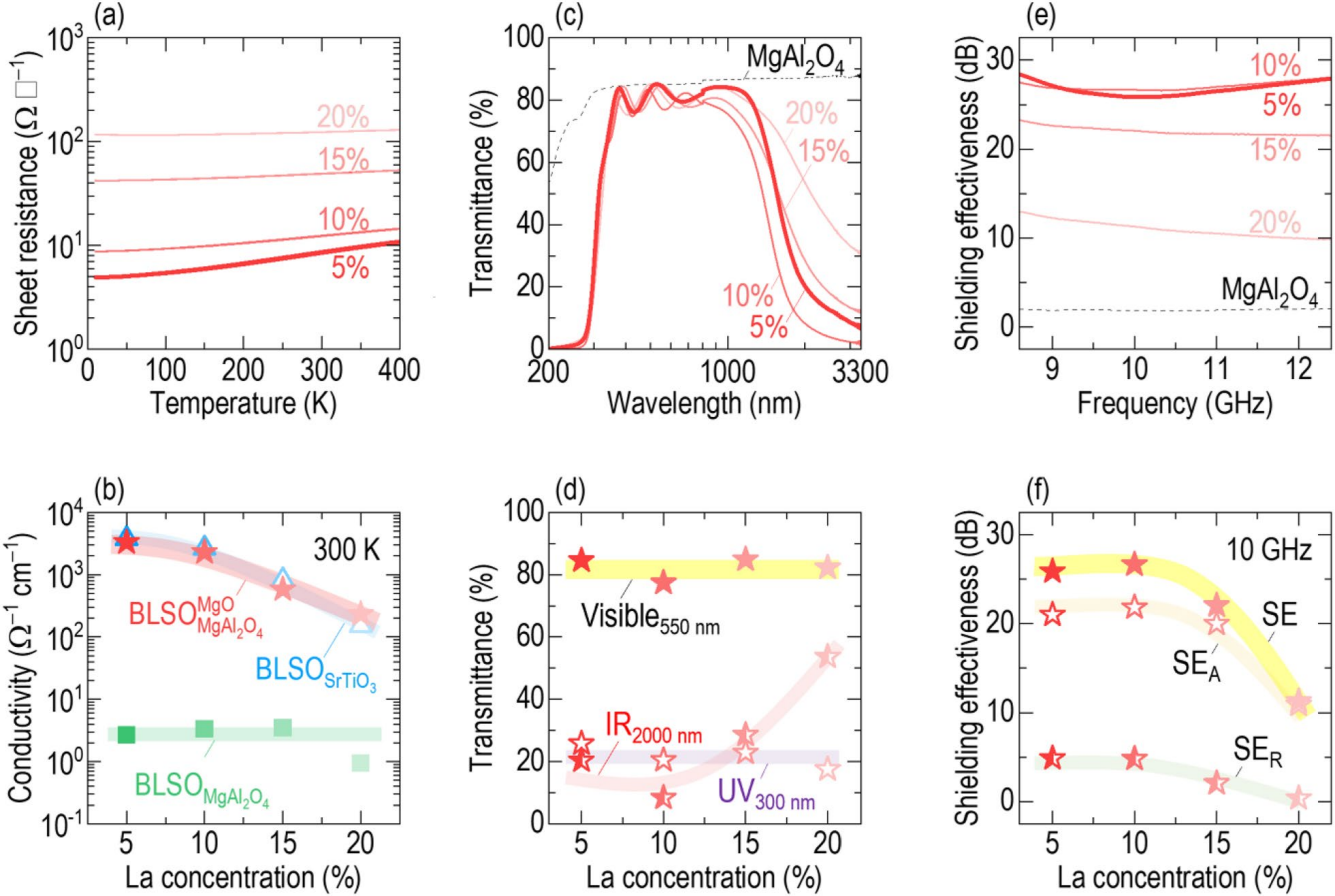
La concentration dependence (5−20%) of the TC-EMS performance of ${\mathrm{BLSO}}_{{\mathrm{MgAl}}_{2}{\mathrm{O}}_{4}}^{\mathrm{MgO}}$. **a** The sheet resistance of ${\mathrm{BLSO}}_{{\mathrm{MgAl}}_{2}{\mathrm{O}}_{4}}^{\mathrm{MgO}}$ decreases with a decrease in the La concentration. **b** The conductivity of ${\mathrm{BLSO}}_{{\mathrm{MgAl}}_{2}{\mathrm{O}}_{4}}^{\mathrm{MgO}}$ and ${\mathrm{BLSO}}_{{\mathrm{SrTiO}}_{3}}$ is maximized in Ba_0.95_La_0.05_SnO_3_ whereas that of ${\mathrm{BLSO}}_{{\mathrm{MgAl}}_{2}{\mathrm{O}}_{4}}$ is invariant. **c** The infrared transmittance of ${\mathrm{BLSO}}_{{\mathrm{MgAl}}_{2}{\mathrm{O}}_{4}}^{\mathrm{MgO}}$ decreases with a decrease in La concentration, but the ultraviolet–visible transmittance does not change; this is shown more clearly in (**d**). **e** The SE of ${\mathrm{BLSO}}_{{\mathrm{MgAl}}_{2}{\mathrm{O}}_{4}}^{\mathrm{MgO}}$ increases with a decrease in La concentration. **f** The SE_A_ and SE_R_ at 10 GHz also increase

We next investigated the effects of the La concentration in ${\mathrm{BLSO}}_{{\mathrm{MgAl}}_{2}{\mathrm{O}}_{4}}^{\mathrm{MgO}}$. Motivated by reports that the conductivity of BLSO films and single crystals is dependent on the La concentration [13, 16], we measured the sheet resistance of ${\mathrm{BLSO}}_{{\mathrm{MgAl}}_{2}{\mathrm{O}}_{4}}^{\mathrm{MgO}}$ with different La concentrations (5–20%) (Fig. 4a). The sheet resistance decreased with the La concentration. We compared the La concentration dependence of conductivity at room temperature among ${\mathrm{BLSO}}_{{\mathrm{MgAl}}_{2}{\mathrm{O}}_{4}}^{\mathrm{MgO}}$, ${\mathrm{BLSO}}_{{\mathrm{MgAl}}_{2}{\mathrm{O}}_{4}}$, and ${\mathrm{BLSO}}_{{\mathrm{SrTiO}}_{3}}$ (Fig. 4b) (Additional file 1: Figure S1 for the La concentration x dependence of the sheet resistance and Additional file 1: Figures S5–7 for the XRD *θ−*2*θ* and *ω* scans). The conductivity of ${\mathrm{BLSO}}_{{\mathrm{MgAl}}_{2}{\mathrm{O}}_{4}}^{\mathrm{MgO}}$ and ${\mathrm{BLSO}}_{{\mathrm{SrTiO}}_{3}}$ decreased with an increase in La concentration; 5%-doped films had the highest conductivity. This optimal La concentration was consistent with 5–10% of La-doped [16], Gd-doped [17], Nb-doped [19], and Ta-doped [20] BaSnO_3_ films. In contrast, the conductivity of ${\mathrm{BLSO}}_{{\mathrm{MgAl}}_{2}{\mathrm{O}}_{4}}$ was insensitive to changes in La concentration and comparable to that of 0.1–1 Ω^−1^ cm^−1^ polycrystals [13]. Irrespective of the La concentration, the templated epitaxy provided by the MgO layer efficiently reduced the sheet resistance of the BLSO films on MgAl_2_O_4_ to near that of ${\mathrm{BLSO}}_{{\mathrm{SrTiO}}_{3}}$.

**Fig. 5:**
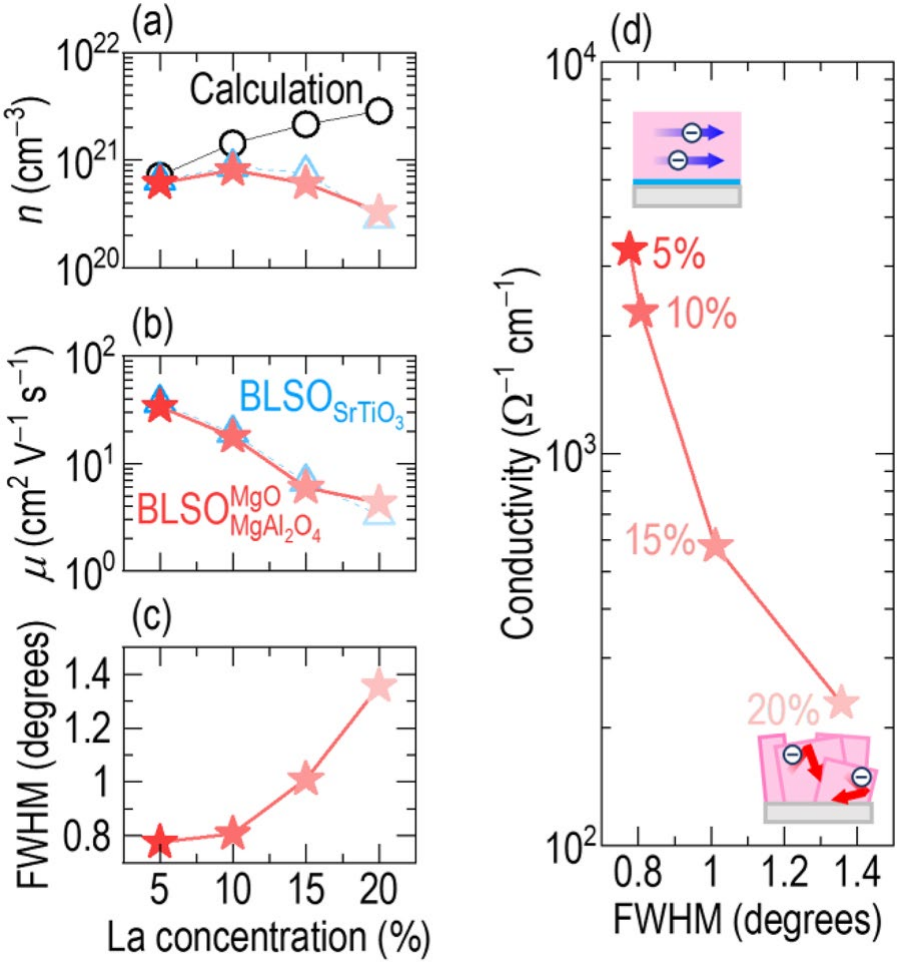
Positive correlation between the conductivity and crystallinity of ${\mathrm{BLSO}}_{{\mathrm{MgAl}}_{2}{\mathrm{O}}_{4}}^{\mathrm{MgO}}$. There is a decrease of (**a**) carrier density and (**b**) mobility with an increase of La concentration in both ${\mathrm{BLSO}}_{{\mathrm{MgAl}}_{2}{\mathrm{O}}_{4}}^{\mathrm{MgO}}$ and ${\mathrm{BLSO}}_{{\mathrm{SrTiO}}_{3}},$ most clearly observed above 10% La doping. **c** At the same time, there is an increase in FWHM of the (002)BLSO XRD peak with La concentration for ${\mathrm{BLSO}}_{{\mathrm{MgAl}}_{2}{\mathrm{O}}_{4}}^{\mathrm{MgO}}$. **d** Overall, there is a decrease in conductivity with an increase in FWHM of the (002)BLSO XRD peak in the ${\mathrm{BLSO}}_{{\mathrm{MgAl}}_{2}{\mathrm{O}}_{4}}^{\mathrm{MgO}}$ films. The 5%-${\mathrm{BLSO}}_{{\mathrm{MgAl}}_{2}{\mathrm{O}}_{4}}^{\mathrm{MgO}}$ film shows the lowest level of electron scattering and highest conductivity

Figure 4c shows the La concentration dependence of the ${\mathrm{BLSO}}_{{\mathrm{MgAl}}_{2}{\mathrm{O}}_{4}}^{\mathrm{MgO}}$ transmittance over the wavelength range of 200–3300 nm. The suppression of infrared transmittance was much steeper in the more conductive $5\% - {\text{BLSO}}_{{{\text{MgAl}}_{2} {\text{O}}_{4} }}^{{{\text{MgO}}}}$ film than in the of $20\% - {\text{BLSO}}_{{{\text{MgAl}}_{2} {\text{O}}_{4} }}^{{{\text{MgO}}}}$ film (Additional file 1: Figure S8 for the transmittance of ${\mathrm{BLSO}}_{{\mathrm{SrTiO}}_{3}}$). According to the Drude–Lorentz model, the absorption coefficient reflecting the free electron response above the plasma frequency is proportional to the carrier density [37]. Therefore, the larger absorption coefficient of $5\% - {\text{BLSO}}_{{{\text{MgAl}}_{2} {\text{O}}_{4} }}^{{{\text{MgO}}}}$ (compared to that of $20\% - {\text{BLSO}}_{{{\text{MgAl}}_{2} {\text{O}}_{4} }}^{{{\text{MgO}}}}$) suggests that carrier density decreases with increasing La concentration (see the experiment in Fig. 5a). However, for all ${\mathrm{BLSO}}_{{\mathrm{MgAl}}_{2}{\mathrm{O}}_{4}}^{\mathrm{MgO}}$ films, the fundamental ultraviolet absorption edge occurred near 300 nm and the visible transmittance was almost invariant at ~ 85%. The donor level of *n*-type BLSO is ~ 46 meV below the conduction band [38], which is very shallow that this will allow the thermal energy (~ 25 meV at room temperature) to “smear out” impurity absorption from donor level to conduction band [37]. Therefore, the bandgap of ${\mathrm{BLSO}}_{{\mathrm{MgAl}}_{2}{\mathrm{O}}_{4}}^{\mathrm{MgO}}$ is governed mainly by valence band to conduction band transitions, making the ultraviolet–visible transmittance similar at all La concentrations. Figure 4d shows more clearly that the infrared transmittance of ${\mathrm{BLSO}}_{{\mathrm{MgAl}}_{2}{\mathrm{O}}_{4}}^{\mathrm{MgO}}$ is sensitive to La concentration, while the ultraviolet–visible transmittance is not.

Figure 4e shows the La concentration dependence of the X-band SE of ${\mathrm{BLSO}}_{{\mathrm{MgAl}}_{2}{\mathrm{O}}_{4}}^{\mathrm{MgO}}$ over the frequency range 8.5–12.5 GHz. The SE values decrease with an increase in La concentration, as does the BLSO conductivity (Fig. 4b). Figure 4f shows that SE_A_ and SE_R_ decrease as the La concentration increases (Additional file 1: Figure S9 for the La concentration dependence of the electromagnetic shielding properties). The Simon formula indicates that SE [dB] increases strongly as the electrical conductivity *σ* [Ω^−1^ cm^−1^] rises: $SE={SE}_{\mathrm{R}}+{SE}_{\mathrm{A}}$, where ${SE}_{\mathrm{R}}=50+10\mathrm{log}\left(\frac{\sigma }{f}\right)$, ${SE}_{\mathrm{A}}=1.7t\sqrt{\sigma f}$, *f* [MHz] is the frequency, and *t* [cm] is the thickness of the electrically conductive material [39, 40].

## Affirmative effect of crystallinity for high conductivity in BLSO films

To explore why both conductivity and SE decreased as the La concentration increased, we investigated the dependence of carrier density and mobility on La concentration [37]. Hall measurements (Additional file 1: Figure S10 for the hall coefficients) showed that the carrier density of ${\mathrm{BLSO}}_{{\mathrm{MgAl}}_{2}{\mathrm{O}}_{4}}^{\mathrm{MgO}}$ increased from 4.2 to 14.1 × 10^20^ cm^−^^3^ as the La concentration decreased (stars in Fig. 5a). This same dependence was also observed in the $5\!-\!20\%-{\mathrm{BLSO}}_{{\mathrm{SrTiO}}_{3}}$ films (triangles) as well as in a previous report on ${\mathrm{BLSO}}_{\mathrm{MgO}}$. [16] For comparison, we calculated the theoretical carrier density of Ba_1−*x*_La_*x*_SnO_3_ (7.2–28.7 × 10^20^ cm^−3^) by assuming that all electron(s) generated by La^3+^ doping in a unit cell of Ba^2+^Sn^4+^O_3_ contributes to the electrical transport. The theoretical density should increase with the La concentration (circles) because, in theory, each La^3+^ on a Ba^2+^ ion leads to the formation of an electron for charge compensation. In fact, the carrier concentration initially rises with La doping but then decreases. This is indicative that electron-trapping defects are created for higher La concentrations. This is understood based on the strain induced in the lattice by the smaller La^3+^ on the larger Ba^2+^ site which would reduce the crystallinity of the films. Figure 5c confirms this as the FWHM for the (002) BLSO XRD peak of ${\mathrm{BLSO}}_{{\mathrm{MgAl}}_{2}{\mathrm{O}}_{4}}^{\mathrm{MgO}}$ increases with increasing La concentration, although it is only a moderate below 10% La substitution.

We now turn to the influence of La concentration on carrier mobility (Fig. 5b). A similar trend is observed as for carrier concentration, i.e., a decrease in carrier mobility with La concentration, e.g., from 36.7 cm^2^ V^−1^ s^−1^ (Ba_0.95_La_0.05_SnO_3_) to 4.3 cm^2^ V^−1^ s^−1^ (Ba_0.8_La_0.2_SnO_3_) for both ${\mathrm{BLSO}}_{{\mathrm{MgAl}}_{2}{\mathrm{O}}_{4}}^{\mathrm{MgO}}$ films (stars) and ${\mathrm{BLSO}}_{{\mathrm{SrTiO}}_{3}}$ films (triangles). The fact that the behavior is the same for these different substrates indicates that the mobility is dominated by intragrain scattering and that the grain boundaries are of similar high quality in the ${\mathrm{BLSO}}_{{\mathrm{MgAl}}_{2}{\mathrm{O}}_{4}}^{\mathrm{MgO}}$ films as in the ${\mathrm{BLSO}}_{{\mathrm{SrTiO}}_{3}}$ films. Also, the high mobilities measured for low La concentrations indicate that there is minimal scattering of free electrons for those concentrations. A previous report on ${\mathrm{BLSO}}_{\mathrm{MgO}}$ showed a similar trend [16].

Since resistivity is a product to carrier concentration and mobility, it would be expected that carrier concentration drops sharply with La concentration for higher carrier concentrations (and from the relation shown in Fig. 5c, crystallinity also, at least above 10% La doping). This is seen from Fig. 5d which shows a sharp drop in conductivity in the ${\mathrm{BLSO}}_{{\mathrm{MgAl}}_{2}{\mathrm{O}}_{4}}^{\mathrm{MgO}}$ films above 10%. The maximum conductivity is for the $5\% - {\text{BLSO}}_{{{\text{MgAl}}_{2} {\text{O}}_{4} }}^{{{\text{MgO}}}}$ which is 1000-fold higher compared to $5\%-{\mathrm{BLSO}}_{{\mathrm{MgAl}}_{2}{\mathrm{O}}_{4}}$ without any template layer. It is clear that there is minimal scattering of free electrons and hence minimal high angle grain boundaries in the high crystallinity films of $5\% - {\text{BLSO}}_{{{\text{MgAl}}_{2} {\text{O}}_{4} }}^{{{\text{MgO}}}}$. Hence, the combination of the MgO template layer and the optimal low La doping level of 5%, produces the highly conductive films. The poorer conduction of ${\mathrm{BLSO}}_{{\mathrm{MgAl}}_{2}{\mathrm{O}}_{4}}$ reflects electron trapping/scattering at the many defects of mixed-crystalline films, as also reported for polycrystalline specimens [13]. However, epitaxial ${\mathrm{BLSO}}_{{\mathrm{MgAl}}_{2}{\mathrm{O}}_{4}}^{\mathrm{MgO}}$ contains few defects, which promote itinerant electron transport. The insets in Fig. 5d show schematically the correlation between suppressed electron scattering and enhanced crystallinity (top image) and vice versa (bottom image).

## Conclusions

We achieved single-crystal-level transparent conduction of Ba_0.95_La_0.05_SnO_3_ epitaxial films on (001) MgAl_2_O_4_ using a MgO template layer. The epitaxial films exhibited lower sheet resistance by three orders of magnitude than Ba_0.95_La_0.05_SnO_3_ films directly grown on MgAl_2_O_4_. We found that 5% La-doping was optimal; the sheet resistance became close to the single-crystal level because of minimal trapping/scattering of free electrons. The use of large and inexpensive MgAl_2_O_4_ wafer substrates guarantees high ultraviolet-visible transmittance (> 85%), which is rarely achieved in most previous studies of epitaxial BLSO films on expensive SrTiO_3_. The electromagnetic SE was as high as ~ 25.9 dB in the X-band. Thus, the conducting, transparent, and electromagnetic shielding properties of BLSO films outperform those of Sn-doped In_2_O_3_ and SrMoO_3_. Given the chemical/thermal/mechanical stability and economic benefits of MgAl_2_O_4_, the single-crystal-level properties of BLSO films on MgAl_2_O_4_ will be useful not only for electromagnetic shielding transparent conductors but also invisible circuitry, smart windows, and solar-energy harvesting.

## Experimental section

### ***Templated epitaxy of La-doped BaSnO***_***3***_*** (BLSO) epitaxial films***

We used pulsed laser epitaxy to deposit 440-nm-thick BLSO films on (001)-oriented MgAl_2_O_4_ with a MgO template layer. To deposit the film and template layer, we ablated Ba_1−*x*_La_*x*_SnO_3_ (*x* = 0.05, 0.1, 0.15, and 0.2) and MgO pellets using an excimer laser (IPEX-760; LightMachinery Inc.) operating at a wavelength of 248 nm, intensity of 1.5 J cm^–2^, and repetition rate of 10 Hz. We heated the substrates to 750 °C using a lamp heater. For BLSO deposition, we maintained an oxygen partial pressure of 75 mTorr using a mass flow controller. However, we used 10 mTorr for MgO growth because the MgO diffraction peaks in the XRD *θ−*2*θ* scan disappeared when films were deposited at 75 mTorr. For comparison, we deposited BLSO films on (001) MgAl_2_O_4_ without the template layer and deposited epitaxial films on (001) SrTiO_3_.

### Characterization of structural properties

We investigated structural properties using a four-circle, high-resolution X-ray diffractometer (Empyrean; PANalytical) emitting Cu radiation at a wavelength of 1.54 Å. We acquired cross-sectional images with a transmission electron microscope (HF-3300; Hitachi) operating at 300 kV with a lattice resolution of at least 1 Å. Fast Fourier transformation was performed using Digital Micrograph software (Gatan Inc.). Energy-dispersive X-ray spectroscopy was used to study the microstructures and elemental distributions of the film and template layer. An atomic force microscope (XE7; Park Systems) operating in contact mode was used to obtain surface images and roughness values; the scan area and rate were 2 × 2 μm^2^ and 0.3 Hz, respectively.

### Measurement of transparent conducting properties

To investigate the transport properties, we deposited four Pt pads in van der Pauw geometry on film surfaces via direct-current magnetron sputtering. Using a physical property measurement system (Quantum Design Inc.), we measured the resistance (< 10 MΩ) under an applied current upon cooling and subsequent heating over the temperature range 10–400 K. We calculated the sheet resistance by multiplying the measured resistance by the geometric factor (2.5) of the films [41]. We derived Hall measurements in a magnetic field ranging from −4 to 4 T at 300 K to determine carrier density and mobility. To directly measure transmittance, we examined films grown on double-sided polished substrates using the transmission mode of an ultraviolet–visible near-infrared spectrophotometer over the wavelength range 175–3300 nm (Cary 5000; Agilent Technologies).

### Measurement of electromagnetic shielding effectiveness

We used a network analyzer (N5222A; Agilent Technologies) to measure SE in the two-coaxial transmission line configuration. We grew films on double-sided, polished (001) MgAl_2_O_4_ substrates (area: 22.8 × 10.1 mm^2^; thickness: 0.5 mm). Each sample was positioned between two waveguides when measuring the *S* parameters (*S*_11_ and *S*_21_) using electromagnetic waves emitted from port 1. *S*_11_ was determined by detecting the reflected wave at port 1. *S*_21_ was acquired by detecting the transmitted wave (i.e., the wave that passed through the film and MgAl_2_O_4_ substrate) at port 2. The total SE was the sum of the absorption (${\mathrm{SE}}_{\mathrm{A}}=10\mathrm{log}\frac{1-{\left|{S}_{11}\right|}^{2}}{{\left|{S}_{21}\right|}^{2}}$) and reflection (${\mathrm{SE}}_{\mathrm{R}}=10\mathrm{log}\frac{1}{1-{\left|{S}_{11}\right|}^{2}}$) components [1, 5]. As SE_A_ was higher than 10 dB, we ignored shielding by multiple reflections [3].

### Supplementary Information

**Additional file 1.**

## Data Availability

The data supporting the findings of this study are available from the corresponding author upon reasonable request.
