# Supplementary material for: La-doped BaSnO3 for electromagnetic shielding transparent conductors
Source: Nano Converg. 2023 Oct 28;10:50. doi: 10.1186/s40580-023-00397-z (PMC10613181; doi:10.1186/s40580-023-00397-z)
Supplement: Supplementary file 1 — Additional file 1. [file 40580_2023_397_MOESM1_ESM.docx]

Figures S1−S10

Tables S1, S2

**1. Sheet resistance of Ba1−*x*La*x*SnO3 (BLSO) films**

**
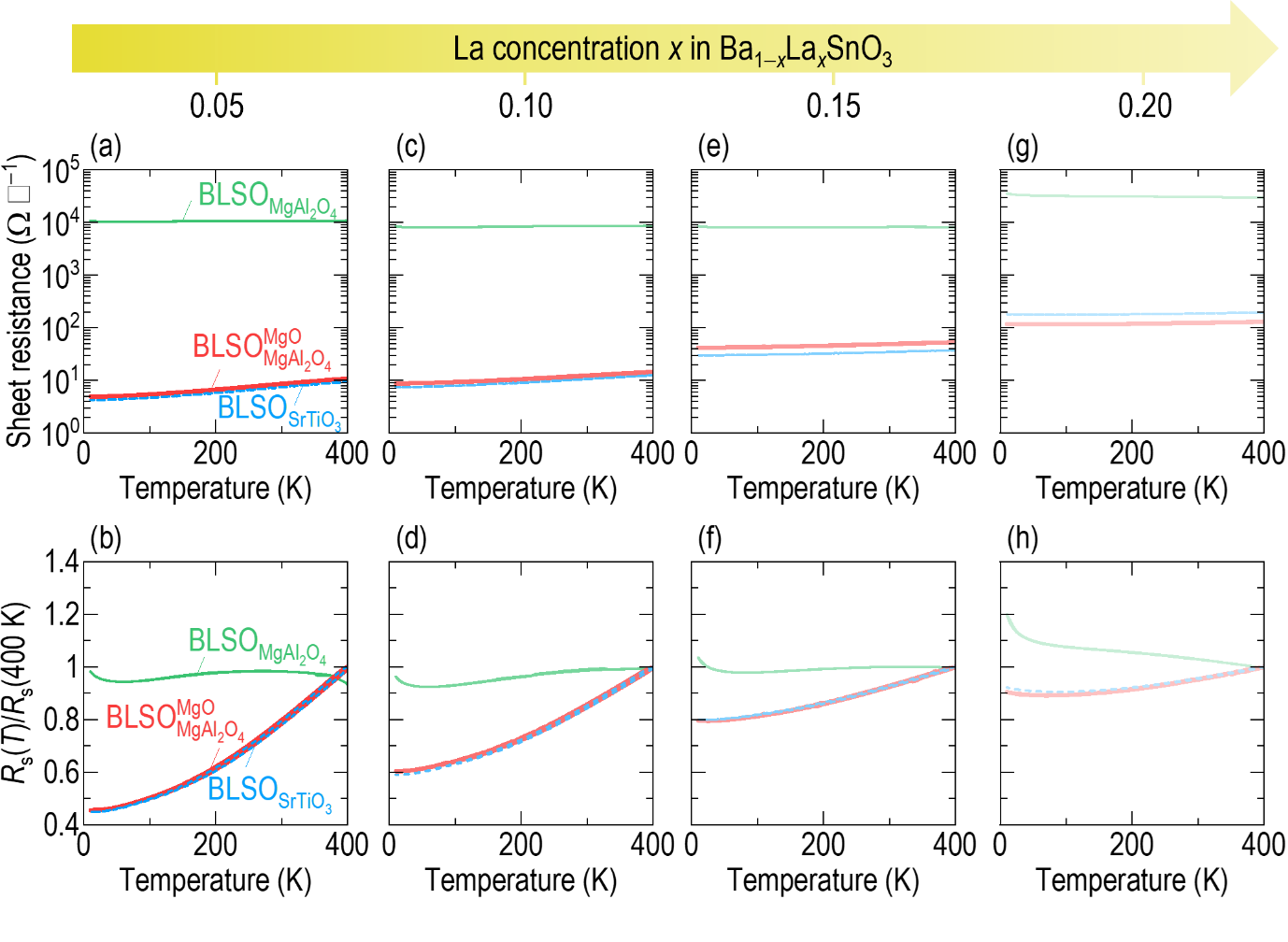
Figure S1.** (a, c, e, g) La concentration *x* dependence of the sheet resistance of , , and . The indicates a Ba1−*x*La*x*SnO3 (BLSO) film grown on a substrate with a template layer. (b, d, f, h) The sheet resistance *R*s(*T*), normalized by the sheet resistance *R*s(400 K) at 400 K. The normalized sheet resistances of and increase with increasing temperature, indicating metallic ground states.

**2. Distribution of conductivity, carrier mobility, and density among ever reports of BLSO**

**
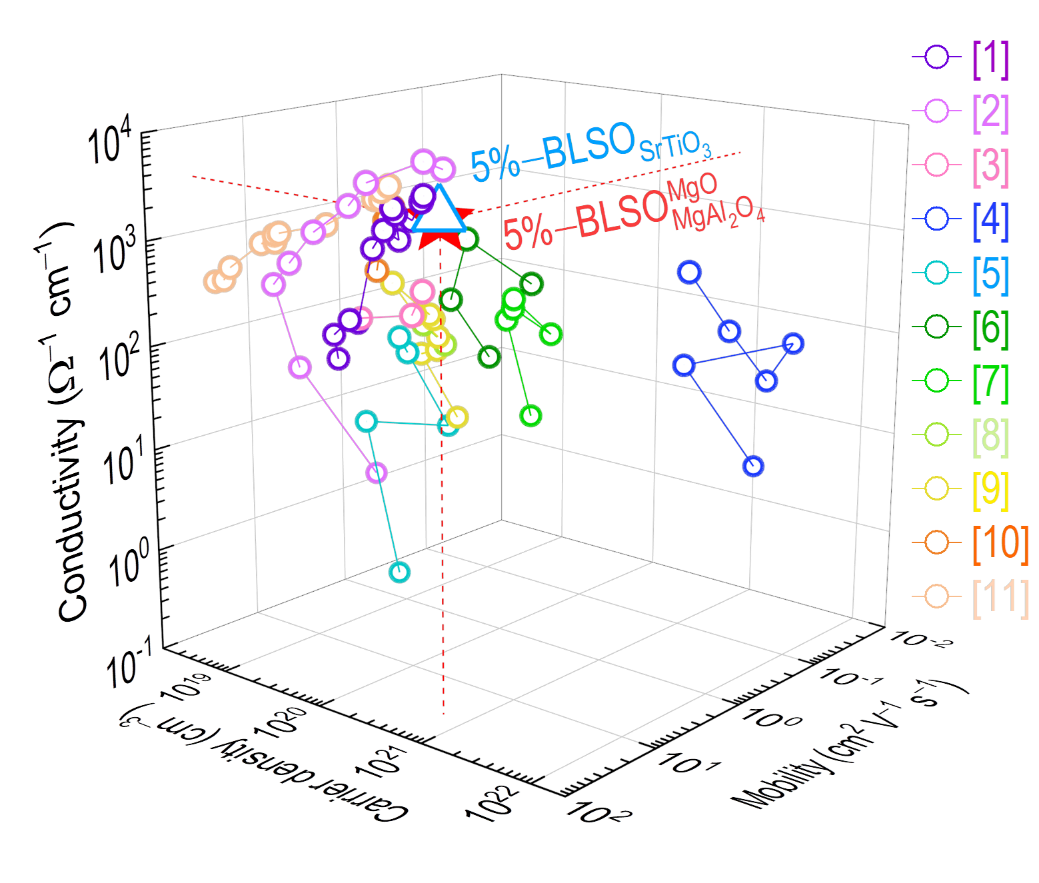
**

**Figure S2.** Comparison of conductivity, carrier mobility, and density among the reports of BLSO. The solid star and open triangle represent our data of and , respectively. Our epitaxial film showed comparable data to those of single crystals, , and , irrespective of the use of industrially practical MgAl2O4 substrates. Since the conductivity, carrier mobility, and density of BLSO are quite sensitive to La concentration, film thickness, and deposition techniques (pulse laser epitaxy, molecular beam epitaxy, sputtering), we believe that the conducting properties of would further increase by their precise optimization.

**Table S1.** Dopant concentration in BaSnO3, film thickness, substrate, and deposition techniques in reports.

| Reference number in Figure S2 | Dopant concentration in BaSnO3 | Film thickness | Substrate | Deposition technique | Reference |
| --- | --- | --- | --- | --- | --- |
| 1 | La, 4 and 7% | 200 nm | SrTiO3 | Pulsed laser deposition (PLD) | *Appl. Phys. Express* **2012,** *5,* 061102 |
| 2 | La | 124 nm | SrTiO3 | Molecular beam epitaxy | *Nat. Commun.* **2017,** *8,* 15167 |
| 3 | La, 1–7% | < 100 nm | SrTiO3 | PLD | *Phys. Rev. B* **2012,** *86,* 165205 |
| 4 | La, 2–20% | 520 nm | MgO | PLD | *Appl. Phys. Lett.* **2012,** *101,* 241901 |
| 5 | Gd, 3–15% | 160 nm | MgO | PLD | *J. Alloy. Compd.* **2015,** *647,* 959 |
| 6 | Nb, 2–15% | 300 nm | MgO | PLD | *J. Alloy. Compd.* **2016,** *680,* 343 |
| 7 | Ta, 3–15% | 300 nm | MgO | PLD | *J. Alloy. Compd.* **2016,** *684,* 125 |
| 8 | La, 8±2% | 320 nm | SrTiO3, SmScO3 | PLD | *Appl. Phys. Lett.* **2014,** *105,* 052104 |
| 9 | La, 8% | – | LaAlO3, SrTiO3, Al2O3 | Chemical solution deposition | *Appl. Phys. Lett.* **2015,** *106,* 101906 |
| 10 | La, 4% | 100 nm | SrTiO3 | PLD | *Appl. Phys. Lett.* **2013,** *102,* 252105 |
| 11 | La, 0.5–4% | 90 nm | BaSnO3 | – | *Appl. Phys. Lett.* **2016,** *108,* 082105 |

**3. Comparison of transparent conductors capable of electromagnetic shielding**

**Table S2.** Resistivity (*ρ* ) and sheet resistance (*R*s,300K) at 300 K, electromagnetic shielding effectiveness at 10 GHz (SE10 GHz) for the X-band, and transmittance at 550 nm (*T*550 nm).

| Materials | *ρ*300 K  (mΩ cm) | *R*s,300 K  (Ω 1) | SE10 GHz  (dB) | *T*550 nm  (%) | References |
| --- | --- | --- | --- | --- | --- |
| Ba0.95La0.05SnO3 films grown on MgAl2O4 with MgO template layers (440 nm) | ~0.3 |  | ~25.9 | ~85 | This study |
| Sn-doped In2O3 films  grown on (0001)Al2O3  (40−360 nm) | ~0.5 |  | 16.1−28.1 | ~80 | *Appl. Surf. Sci.* **2022,** *604,* 154149 |
| SrMoO3 epitaxial films  grown on Al2O3  (45−80 nm) | 0.09 | 10 | 27.3−29.4 | 57−61 | *Adv. Mater. Interfaces* **2022,** *9,* 2200893 |
| Carbon nanotube-polystyrene foam composites |  | − | ~18 | − | *Nano Lett.* **2005,** *5,* 2131 |
| Polyaniline composites  with single-wall carbon  nanotubes or graphene sheets | 50−1,000 | − | 19−29 | − | *J. Phys. D: Appl. Phys.* **2012,** *45*, 235108 |
| Silver nanowire network | − | 15 | ~21 | − | *ACS Appl. Mater. Interfaces* **2017,** *9,* 40857 |
| MXene forms |  | − | 30−70 | − | *Adv. Mater.* **2017,** *29,* 1702367 |
| Graphene sheets |  | − | 12−27 | − | *PNAS* **2018,** *115,* 5359 |
| MXene-CNT |  | − | 90.7 | − | *J. Mater. Chem. A* **2021,** *9,* 24560 |
| MXene-polyvinylidene fluoride |  |  | 42.9 | − | *Compos. Pt. B-Eng.* **2021,** *217,* 108902 |
| Carbon aerogel film |  |  | 41.4 | − | *Carbon* **2021,** *184,* 562 |

**4. BLSO polycrystalline films directly grown on (001)-oriented MgAl2O4**

**
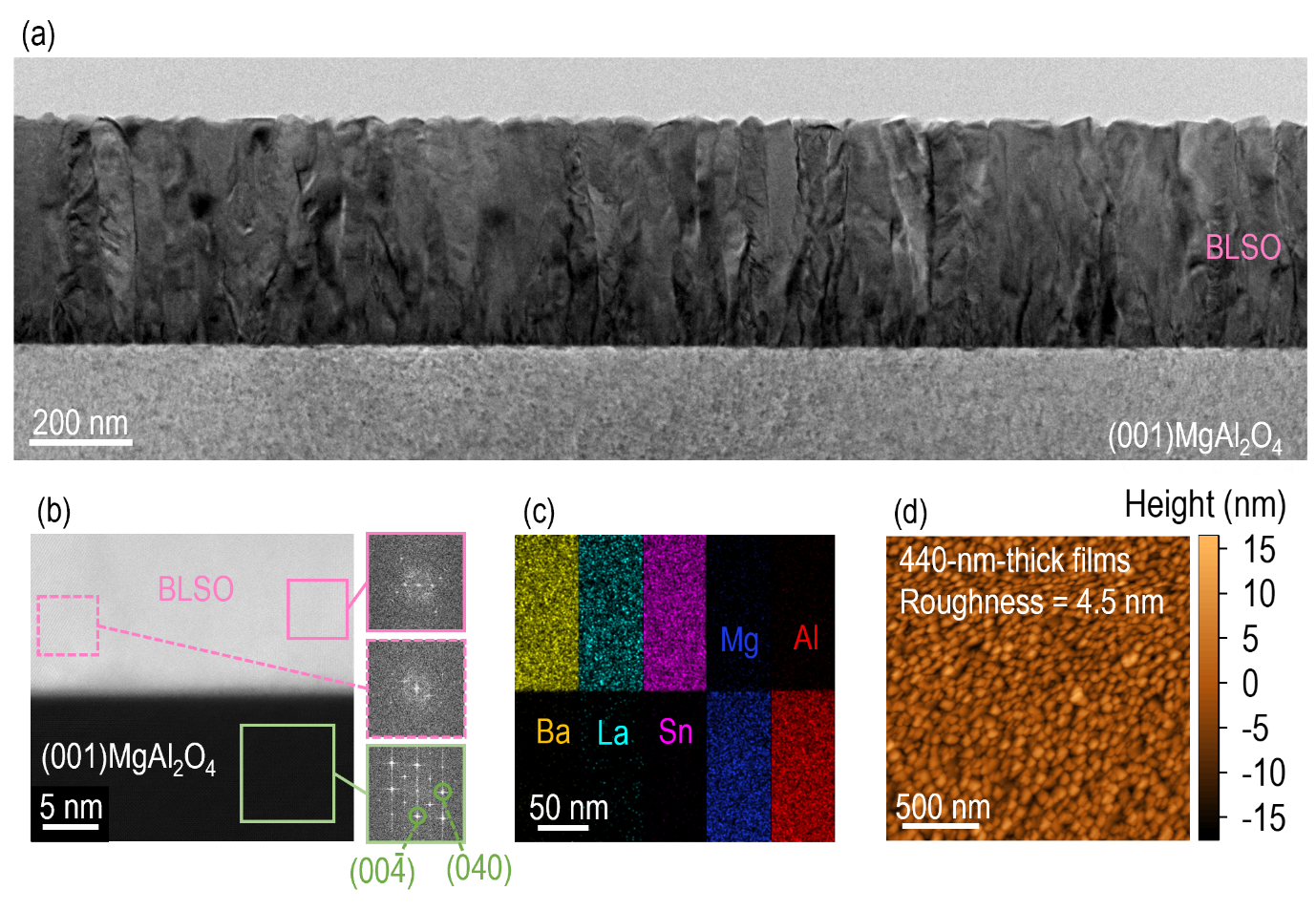
**

**Figure S3.** Nanoscopic investigation of the crystal structure of . (a) The cross-sectional transmission electron microscopic image clearly shows the formation of BLSO film when we directly deposit it on MgAl2O4 substrate. (b) Selected areas show different images of Fast Fourier transform, indicating polycrystalline nature of the BLSO film. (c) Energy-dispersive X-ray spectroscopy reveals negligible intermixing of Ba (yellow), La (green), Sn (purple), Mg (blue), and Al (red) atoms between BLSO film and MgAl2O4 substrate. (d) Atomic force microscopy reveals slightly rough surface (roughness ~4.5 nm) of a 440-nm-thick BLSO film.

**5. Flat surfaces of BLSO epitaxial films grown on (001)-oriented SrTiO3**


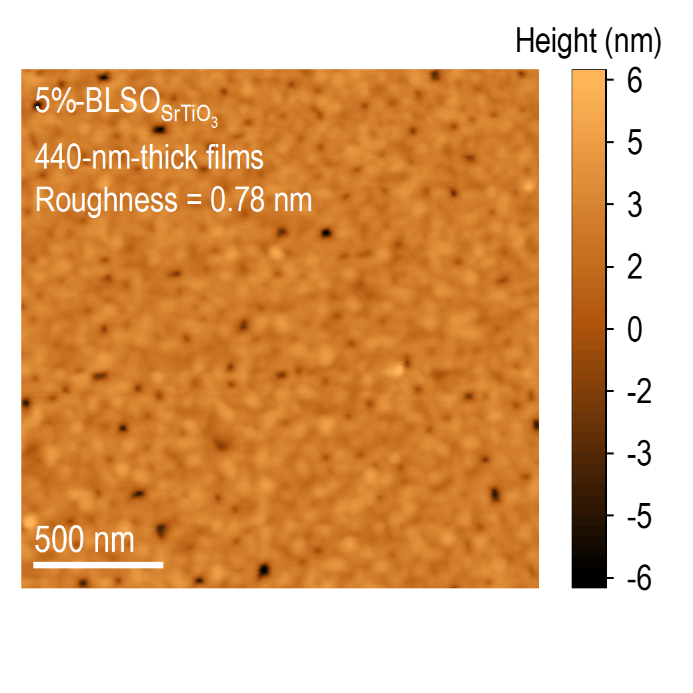


**Figure S4.** The flat surface roughness of 440-nm-thick BLSO epitaxial films grown on (001)-oriented SrTiO3 was ~0.78 nm, taken by atomic force microscopy.

**6. Crystallinity of BLSO films on various substrates**


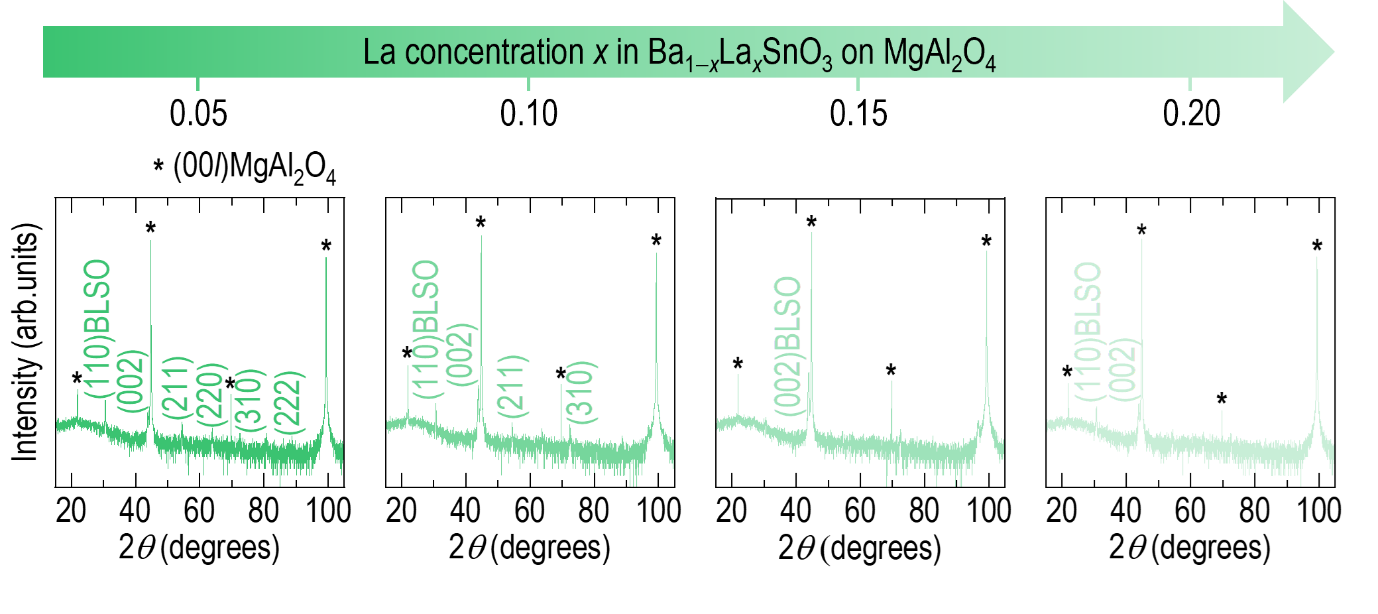


**Figure S5.** X-ray diffraction *θ*−2*θ* scans of poorly crystalline .

**
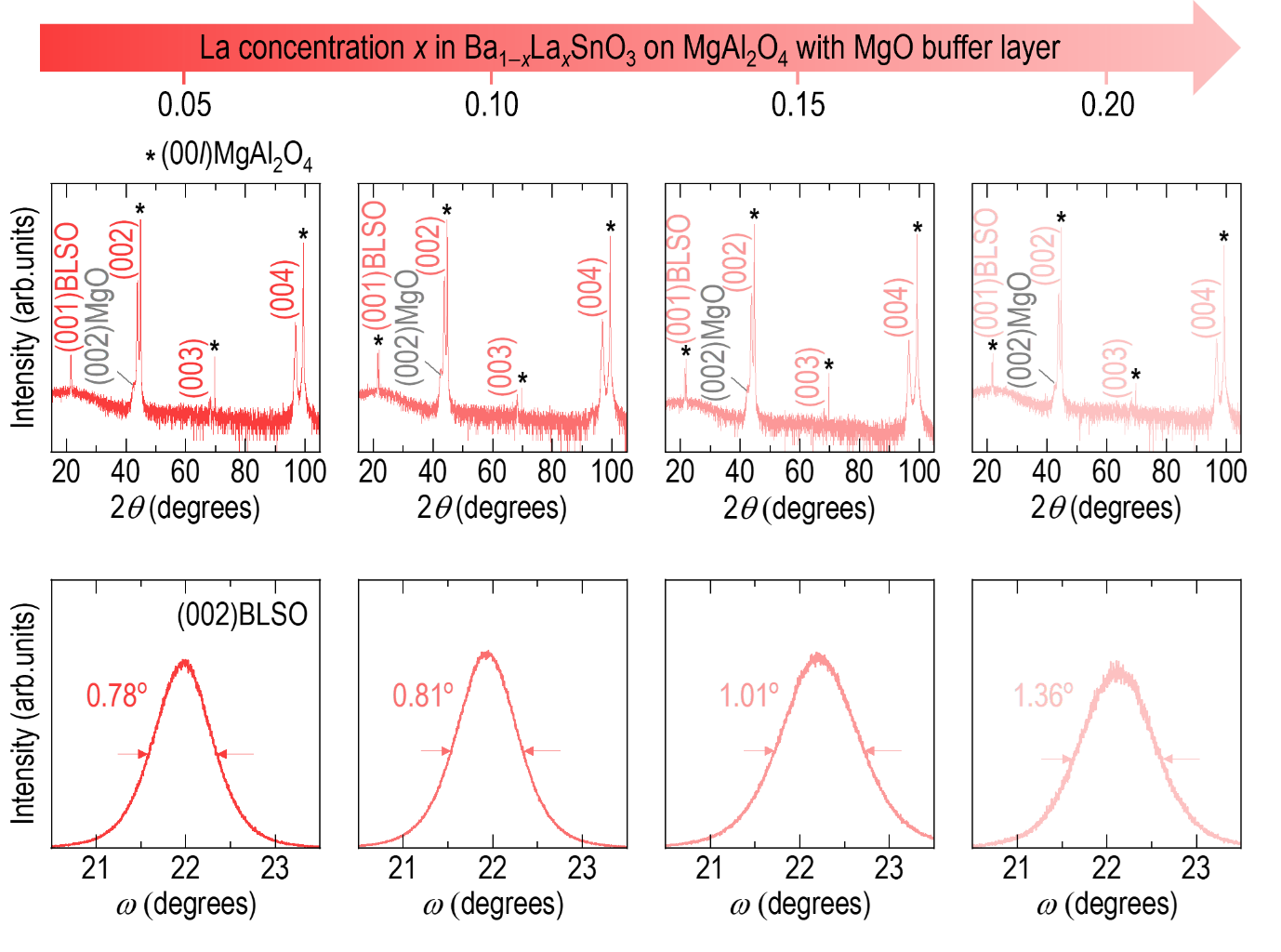
**

**Figure S6.** X-ray diffraction *θ*−2*θ* scans (upper row) and *ω* scans (lower row) of highly crystalline .


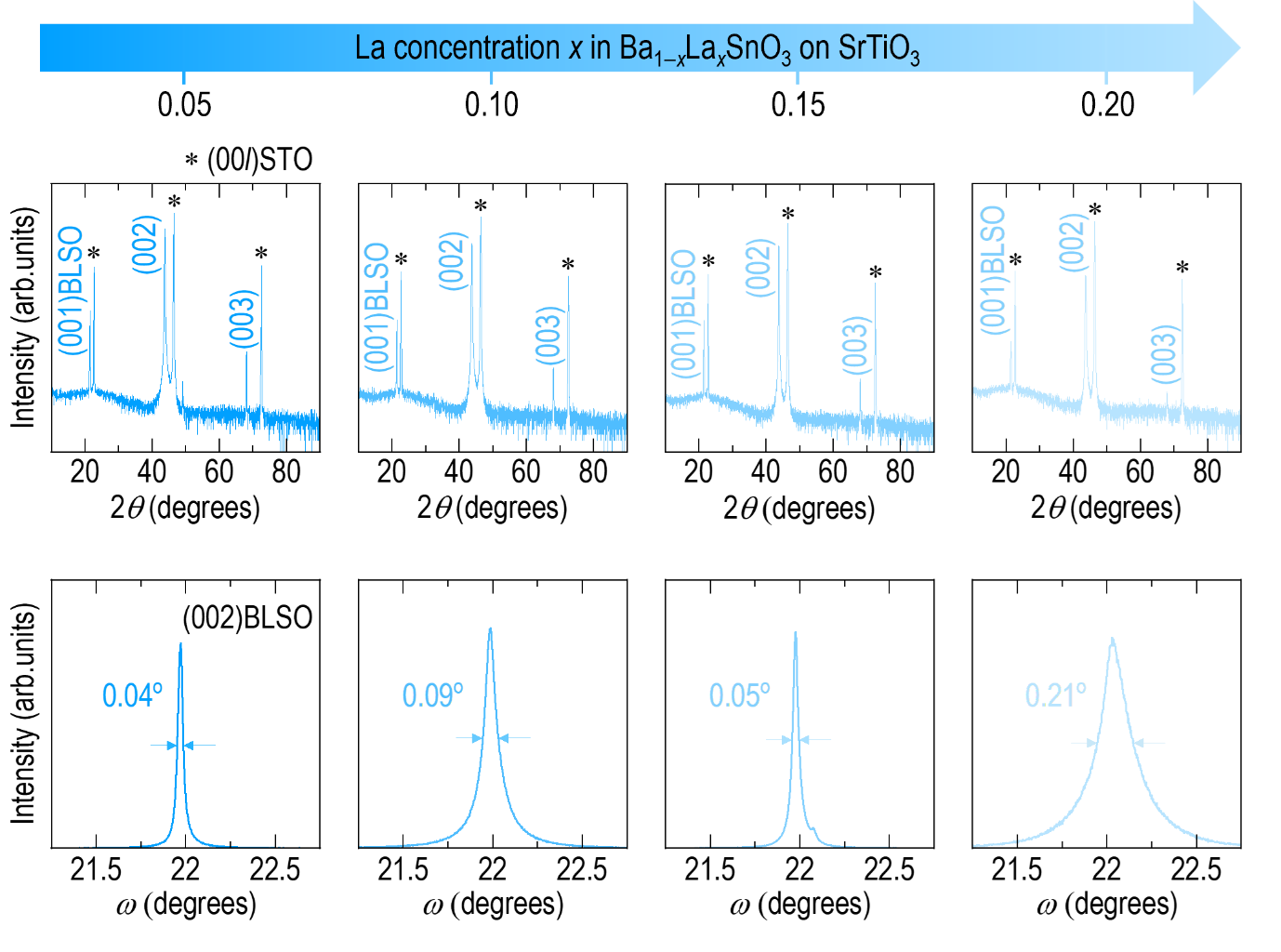


**Figure S7.** X-ray diffraction *θ*−2*θ* scans (upper row) and *ω* scans (lower row) of highly crystalline .

**7. La concentration-dependence of the transmittance of BLSO epitaxial films on (001)SrTiO3**


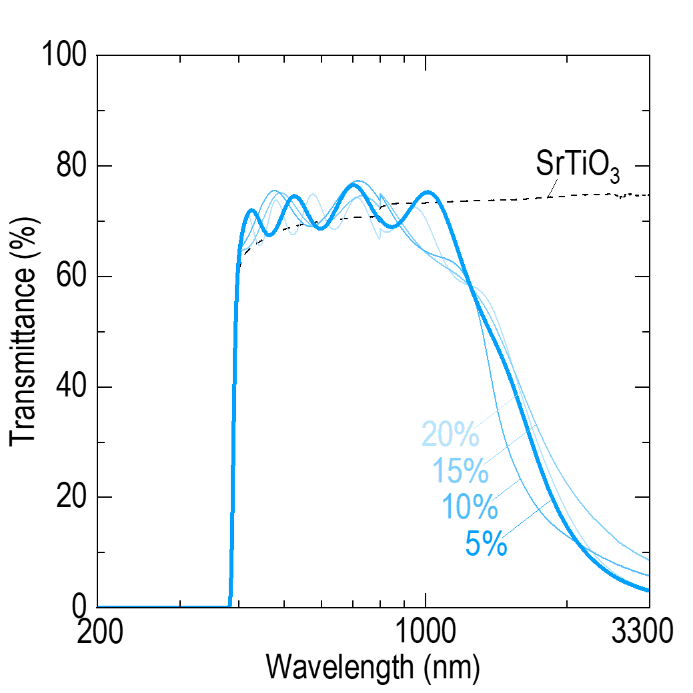


**Figure S8.** Transmittance of BLSO epitaxial films on (001)SrTiO3 according to the La concentration. The infrared transmittance of decreases with the increasing La concentration because the number of free electrons decreases; the visible transmittance does not change with La concentration. The abrupt drop in ultraviolet transmittance is attributable to the smaller bandgap (3.2 eV) of SrTiO3 than the ~4.1 eV of BLSO.

**8. La concentration dependence of the electromagnetic shielding properties of BLSO epitaxial films on (001)MgAl2O4 with an MgO template layer**


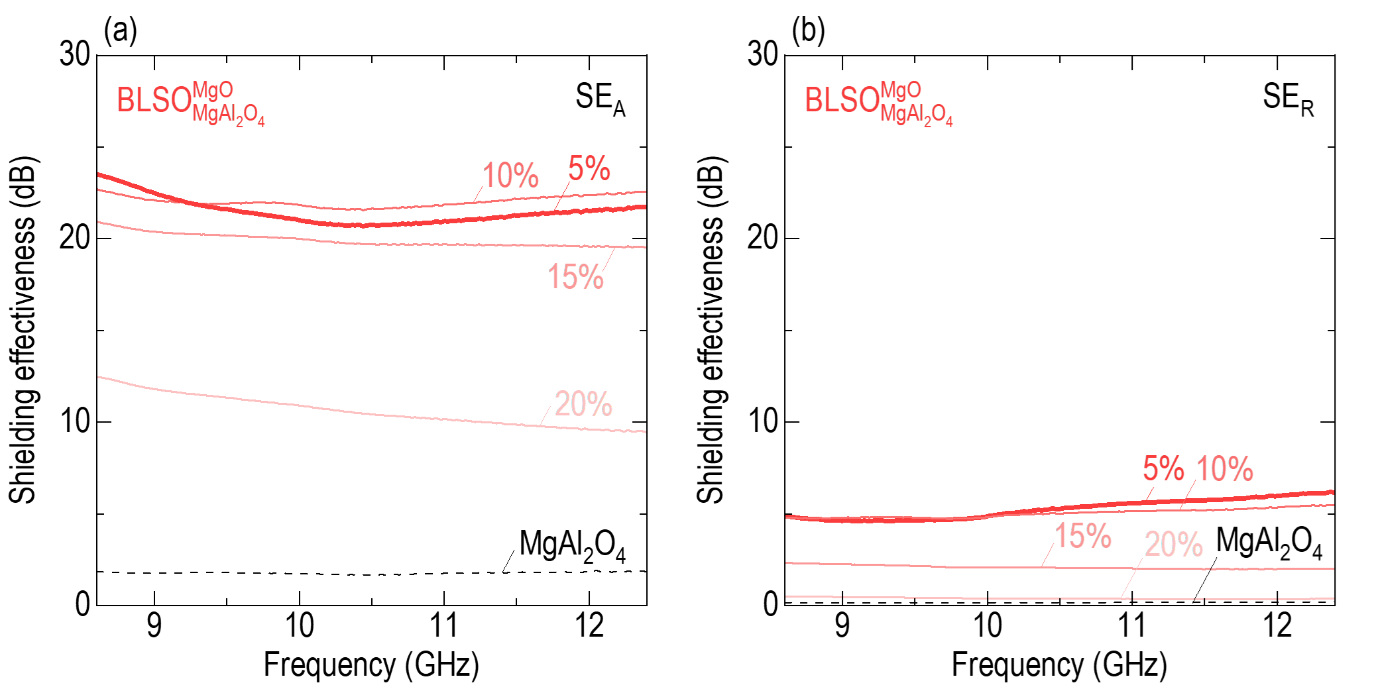


**Figure S9.** Decrease in the (a) SEA and (b) SER of with increase in La concentration, showing the maximum values for 5% La concentration. The SEA and SER represent the wave transmitted through the film and MgAl2O4 substrate, and the wave reflected from the BLSO film, respectively.

**9. Hall measurements of BLSO films**

**
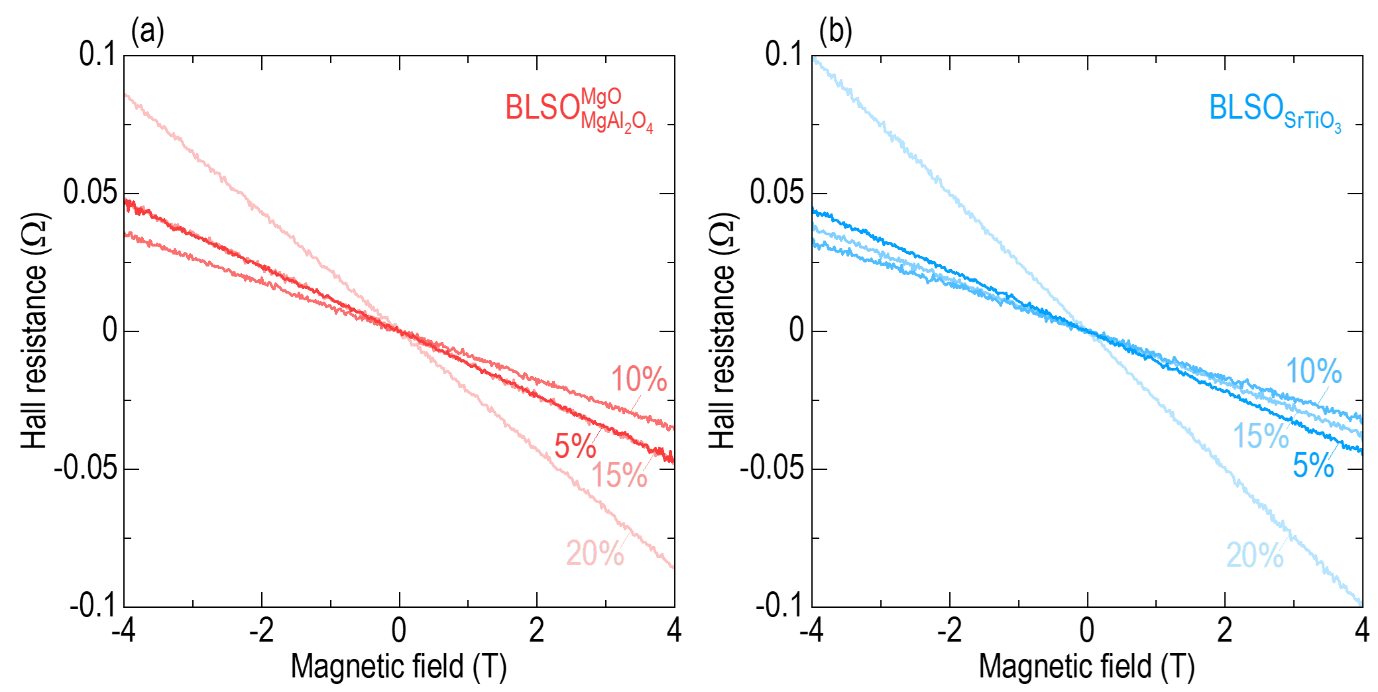
**

**Figure S10.** Hall coefficients of (a) and (b) measured using the four Pt pads in van der Pauw method at 300 K. We quantified the dependence of the Hall resistance on the magnetic field by sweeping the magnetic field strength from −4 to 4 T. The negative slopes indicate that the BLSO epitaxial films have *n*-type semiconducting properties. The slope of a Hall resistance–magnetic field curve is the Hall coefficient. We calculated the carrier density by dividing the Hall coefficient by the elementary charge. Finally, we obtained the mobility by dividing the conductivity by the carrier density and elementary charge.
